# Causes of Loss of Confidence in X-ray Treatment

**Published:** 1906-04

**Authors:** M. B. Hutchins

**Affiliations:** Atlanta, Ga.


ATLANTA

### Journal-Record of Medicine

Successor to Atlanta Medical and Surgical Journal, Established 1855, and Southern Medical Record, Established 1870.

OWNED BY THE ATLANTA MEDICAL JOURNAL CO.

Published Monthly.

VOL. VIII. APRIL, 1906. No. 1.

BERNARD WOLFF, M.D., EDITOR, Nos. 319–20 Prudential.

M. B. HUTCHINS, M.D., BUSINESS MANAGER, 1007–1008 Century Bldg.

E. G. BALLENGER, M.D., ASSOCIATE EDITOR AND ASSISTANT MANAGER.

### ORIGINAL COMMUNICATIONS.

### CAUSES OF LOSS OF CONFIDENCE IN X-RAY TREATMENT.*

BY M. B. HUTCHINS, M.D.,

ATLANTA, GA.

I am aware that papers on X-ray treatment have been, and are, so common that I should possibly apologize for an additional contribution. The subject chosen was suggested by hearing repeatedly remarked by some doctor that he had no faith in this treatment. In a way this loss of faith in X-rays as a therapeutic agent will do good. Five years ago the craze began and soon a great many manufacturers of electric machinery had begun making X-ray outfits. Then the doctors in general work caught the fever and began to buy machines.

Many of the manufacturers have given up this branch and many doctors are finding their early enthusiasm and prospect of riches fading away. The reason is easy to find.

Like every other agent used for the cure of disease it has its

*Read at Joint County Society Meeting, Covington, Ga., March 20, 1906.

LIBRARY
SURGEON GENERAL'S OFFICE
AUG.-21-1907
197226.

limitations, meets its failures. The pendulum of credulous faith swung too far, has swung quite far back, will finally follow a steady course.

It may surprise you to know that some distinguished doctors do not believe in the diphtheria antitoxin. It can't save every case. Does mercury cure every case of syphilis, quinine all cases of malaria, salicylates every case of rheumatism? What is the printed record of the operative treatment of all cancers? It is as bad as or worse than the record of X-rays. Do we cure all of our cases of psoriasis, eczema, acne, hypertrichosis, mycosis fungoides, cutaneous epithelioma and sarcoma with other methods? The practice of medicine is not perfect, the use of no agent can be followed by invariable success.

The abuse of any remedy will bring it into disrepute. But X-rays have been in the limelight for the past few years—the central figure. It was the big thing in treatment as appendicitis is the fashionable disease. A failure with X-rays attracts nearly as much attention as a death from appendicitis, but cancer plaster cases pass as unheeded as the death of a man with a tumor of the brain.

We must, in weighing the value of X-rays, compare their successes and failures fairly. Would you expect me to have as good record in appendicitis work as the men who have perfected the technique of this treatment? Could the oculist get as good results in typhoid fever as you general men? Could you do cataract work as well as they? In estimating results we must consider the training of the operator. Men with no knowledge of skin disease or cancer went into the X-ray business—that is the name—and to their sorrow. They got some good results, but men competent enough in other lines began to have disasters, not only in utter failure to cure but in producing destructive injuries, some even resulting in X-ray burn-cancer.

Deep-seated cancer is incurable. Every man who takes a case should give all parties interested to understand that the treatment is but a hopeful struggle against a hopeless disease.

Time and space are too limited to report kinds of diseased conditions amenable to scientific X-ray treatment. I have done this in another paper.

The operator must know his machine, his tubes and, as far as possible, their variations. Then he must know the pathological conditions he treats, the susceptibility of the different tissues and of different people and be able to distinguish between an X-ray burn and aggravation or progression of the disease.

It seems to me that a doctor has gone a long way towards being safe when he gets to know what he can't do. There is a lot that we can't do with X-rays, there is much that can be done and a great deal that we should never dare try to do.

Above all the use of any means of cure should be governed, not by a hope of success and a mercenary desire of financial improvement, or a love of the spectacular, but by a more than plain honesty, an honesty so clear and pure that not only does the pitiable sufferer receive support by it but that the physician of the tenderest conscience can have no nightmare doubts as to whether some poor victim has been led into false hopes and his purse emptied by deception.

Upon my mind is the weight of depressing evidence of X-ray failures of my own, but failures honestly made on cases in which my hopes were equal to or greater than those of my patients. On the other hand is the record of much good done by this treatment, cures or added months of life and interest in it and the gratitude of some few.

So, gentlemen, let us give X-rays their fair and proper place as a valuable aid in our therapeutics and when tempted to decry them as useless turn to our old friends, mercury, quinine, alcohol, antitoxins and opium and ask if they have ever failed, or if their pathway through the years shows only the flowers of success with no headstones marking failures.

---

When operating upon the ureter for calculus or stricture, avoid undue manipulation; it is important to prevent detachment of the ureter from its bed, if possible.—*American Journal of Surgery.*

